# Incidence and outcomes of contrast-induced nephropathy in adult ICU patients

**DOI:** 10.1186/cc13564

**Published:** 2014-03-17

**Authors:** S Alderson, S Pillai, A Manoras, J Papanikitas, D Lewis, S McKechnie

**Affiliations:** 1 John Radcliffe Hospital, Oxford, UK; 2 Morriston Hospital, Swansea, UK

## Introduction

Critically ill patients cared for in ICUs often require radiological investigation using iodinated contrast agents. Contrast- induced nephropathy (CIN) - a form of acute kidney injury (AKI) - is a complication following the use of such contrast. Although CIN has been thoroughly studied in some populations (for example, those undergoing coronary angiography), it has not been investigated in large numbers of ICU patients [1].

## Methods

We conducted a single-centre retrospective review of the electronic patient records of general adult ICU patients who received iodinated contrast over a 3-year period (2009 to 2011). Our review identified evidence of CIN or AKI post scan (assessed using CIN and KDIGO criteria); and clinical outcomes post scan (renal replacement therapy (RRT), ICU length of stay, mortality). Patients were excluded if: they had received pre-scan RRT; they did not have pre/post-scan creatinine measurements. Univariate analyses investigated the relationship between CIN or AKI and ICU outcomes (use of RRT post scan, ICU mortality and ICU length of stay), using chi-squared tests for categorical outcomes and nonparametric tests for continuous outcomes.

## Results

A total of 479 scans involving 331 patients were included. In total, 303 (63%) scans involved males, median age 61 (IQR 46 to 71) years, 119 (25%) diabetic, with median pre-scan eGFR of 85 (38 to 113) ml/minute/1.73 m^2^. Scans occurred a median of 2.9 (0.8 to 8.8) days from admission. A total of 266 (56%) scans were associated with CIN (grade 0, *n *= 167; grade 1, *n *= 39; grade 2, *n *= 60). Clinical outcomes were significantly worse in patients developing higher grades of CIN (increased use of RRT post scan, *P *= 0.02; increased ICU length of stay, *P *= 0.04; increased ICU mortality, *P *= 0.01). Ninety-five (20%) scans were associated with AKI (stage 1, *n *= 45; stage 2, *n *= 13; stage 3, *n *= 37). Clinical outcomes were again significantly worse in patients developing AKI (increased use of RRT post scan, *P *< 0.001; increased ICU mortality *P *= 0.01).

## Conclusion

Renal impairment/injury is common in adult ICU patients undergoing investigations using iodinated contrast and the incidence varies depending upon the classification used. Whichever classification is used, patients developing CIN/AKI following contrast administration have poorer clinical outcomes than patients who do not.

## References

[B1] Lameire Contrast-induced acute kidney injury and renal support for acute kidney injury: a KDIGO summary (Part 2) Crit Care 2013 17 205 10.1186/cc11455 23394215 PMC4056805

